# Isolation, characterization and PCR multiplexing of microsatellite loci for a mite crop pest, *Tetranychus urticae* (Acari: Tetranychidae)

**DOI:** 10.1186/s13104-015-1194-9

**Published:** 2015-06-17

**Authors:** Laure Sauné, Philippe Auger, Alain Migeon, Jean-Emmanuel Longueville, Simon Fellous, Maria Navajas

**Affiliations:** INRA, UMR1062 CBGP, Campus International de Baillarguet, CS 30016, 34988 Montferrier-sur-lez Cedex, France

**Keywords:** *Tetranychus urticae*, Spider mite, Microsatellite, Multiplex PCR

## Abstract

**Background:**

*Tetranychus urticae* is a highly polyphagous species with a cosmopolitan distribution that has the status of pest in more than 100 economically significant crops all over the world. Despite a number of previous efforts to isolate genetic markers, only a reduced set of microsatellite loci has been published. Taking advantage of the whole genome sequence of *T. urticae* that recently became available; we isolated and characterized a new set of microsatellite loci and tested the level of polymorphism across populations originating from a wide geographical area.

**Results:**

A total of 42 microsatellite sequences widespread in the *T. urticae* genome were identified, the exact position in the genome recorded, and PCR amplification of microsatellite loci tested with primers defined here. Fourteen loci showed unambiguous genotype patterns and were further characterized. Three multiplex polymerase chain reaction sets were optimized in order to genotype a total of 24 polymorphic loci, including 10 previously published *Tetranychus*-specific loci. The microsatellite kits successfully amplified 686 individuals from 60 field populations for which we assessed the level of genetic diversity. The number of alleles per locus ranged from 3 to 16 and the expected heterozygosity values ranged from 0.12 to 0.81. Most of the loci displayed a significant excess of homozygous and did not model the Hardy–Weinberg equilibrium. This can be explained by the arrhenotokous mode of reproduction of *T. urticae*.

**Conclusions:**

These primers represent a valuable resource for robust studies on the genetic structure, dispersal and population biology of *T. urticae*, that can be used in managing this destructive agricultural pest.

## Findings

*Tetranychus urticae* (the two spotted spider mite) is a cosmopolitan and highly polyphagous species. This mite has been reported from about 1059 host plants and is a major pest for 100 crops [1]. Despite the worldwide distribution and high agricultural relevance of the species, the extent of its genetic diversity still lacks of information. The evolutionary history of *T. urticae*, while only partially explored, indicates high biodiversity. Analyses based on MtDNA COI sequences split *T. urticae* populations in two clearly separated clades with 5% of nucleotide divergence [2]. This uncovered diversity and the broad relevance of the species, has motivated intensive efforts to isolate fine-resolution markers for population studies. Early screening of genomic libraries indicated an under-representation of microsatellite sequences in the mite genome [3]. Subsequent efforts to determine genetic markers led to the isolation and use of a reduced number of microsatellites [4, 5] and motivated population genetic studies. While the isolation by geographical distance had appeared as a major factor of population structure [6, 7]. The host plants where the mite develops seems to also have some influence. Genetic data from mites collected on citrus groves suggests some population differentiation between individuals collected on trees and weeds [8], while high level of dispersion among apple orchards tend to reduce genetic differentiation [9]. Genetic markers have also helped to further clarify taxonomic issues as the status of the red and green forms of *T. urticae* [6]. However, due to the limited number of markers used so far (usually five), the information remains limited to clearly understand the genetic diversity of the species and spots the need for an increased number of fine scale markers.

The whole genome sequence of *T*. *urticae* became recently available [10], facilitating the detection of additional microsatellite sequences. Some attempts to use cross-hybridizing primers to amplify *Tetranychus* species close to *T. urticae* have also been published [11]. The isolation and characterisation of a new set of microsatellite markers reported in this paper represents a valuable resource to deeper understand the biology of *T. urticae*. Estimates of gene flow and then of movements of individuals, to predict dissemination of genes involved in pesticide resistance, or to assess the impact of host plants as reservoirs (e.g. [8]), are a few examples of the questions that can be addressed using fine-scale markers, which should also help to manage mite populations of this important crop pest.

The isolation and characterization of the new set of primers was based on sequences of the *T. urticae* genome. All sequencing reads were collected with standard Sanger sequencing protocols on ABI 3730XL capillary sequencing machines at the Department of Energy Joint Genome Institute (DOE-JGI; Walnut Creek, CA, USA). The final assembly contains 640 scaffolds that cover 89.6 Mb of the genome with a contig L50 of 212.8 kb and a scaffold L50 of 3.0 Mb [10]. A total of 8,435 regions ranging from 413 to 720 bp were identified. Among them 546 and 1389 included di and tri-nucleotide repeats respectively (as analysed using QDD [12]). The exact position of the selected sequences was recorded according to the whole genome sequence from individual scaffolds of the *T. urticae* (London strain) genome available through GenBank under accession numbers HE587301 to HE587940 (see also http://bioinformatics.psb.ugent.be/orcae/overview/Tetur).

Primers design was done with the program QDD [12]. Sequences longer than 80 base pairs (bp) and containing perfect microsatellites of at least five repetitions for any motif of 2–6 nucleotides were selected for further analyses. PCR primers were designed using QDD with the following stringent criteria: (1) target microsatellites had at least five repetitions, (2) length of PCR products were between 90 and 300 bp, (3) flanking regions did not contain either any homopolymer stretch of more than four bases or any di-hexa base pair motifs of more than two repetitions, (4) annealing temperatures of primer pairs were optimized to 55°C and (5) microsatellites were not compound or interrupted. We selected a subset (n = 54) of sequences for which primers were designed for PCR amplification.

Total DNA was extracted from adult female mites with DNeasy 96 Blood and Tissue kit (Qiagen®). PCR amplifications were initially performed on eight individuals for each of the 54 primer pairs in a total volume of 10 μL containing 2 μL of DNA extract using the Multiplex PCR Kit (Qiagen®). Thermocycling was performed on a Mastercycler® gradient (Eppendorf) with the following protocol: 95°C for 15 min, followed by 35 cycles (94°C for 30 s, 55°C for 90 s, 72°C for 1 min), and 60°C for 30 min. Out of the 54 primer pairs, 42 displayed clear PCR products on agarose gel electrophoresis, i.e. discrete single bands or at most two bands when there were large differences in size between alleles. The remaining 12 primer pairs either did not amplify in some of the 8 individuals or produced multiple bands or smears. The loci were amplified separately using forward primers labelled with the fluorescent dyes 6-FAM, PET, NED or VIC (Applied Biosystems). The PCR products were visualized using an ABI 3130XL Genetic Analyzer (Applied Biosystems). Allele sizes were scored against an internal GeneScan-500 LIZ® Size Standard (Applied Biosystems) and genotypes obtained using GeneMapper® 3.7 (Applied Biosystems).

Among the 42 screened markers, 14 showed unambiguous genotype patterns and were kept and amplified into three PCR multiplex kits in combination with 10 primers pairs previously described [9, 4, 13] (Table 1). The three multiplex sets were tested using the amplification protocol described above.

**Table 1:** Characterization and levels of variability at 24 microsatellite loci of *Tetranychus urticae*

Locus	Motif	Primer sequence (5′–3′)	Scaffold number	Number of set	Dye	GenBank accession No	Size range	Na	*H*o	*H*e
TuLS14	(ATG)7	F: GCAAATGAAGCTTACCAATTA	17	2	VIC	KJ545959	191–210	7	0.2	0.60
		R: TAAAGGTTTGGCAGTTCAGT								
TuLS16	(CAT)10	F: AATTGCTTATCACCCACATC	21	2	PET	KJ545960	186–228	16	0.39	0.79
		R: TTAGTTGCTTGTTGAGCAGA								
TuLS17	(ATG)6	F: TCTTCGTTCGATAGCTTTTC	23	2	FAM	KJ545961	192–207	6	0.03	0.55
		R: TCCTCAGGTATATCAGGTGG								
TuLS19	(TG)6	F CAAAAGTTGGACATTTCAGG	24	2	NED	KJ545962	195–211	8	0.28	0.71
		R: TCCTTCCACAGTCAATATCC								
TuLS20	(TTG)6	F: AAGCTGGATTCATAGAAGCA	27	1	PET	KJ545963	212–218	3	0.16	0.38
		R: AAATTAATTCAGCCTCGTCA								
TuLS22	(TG)6	F: GCAATCGTTTGTTTTCATTT	33	1	NED	KJ545964	191–203	5	0.17	0.43
		R: TCACAATTGATGATGCTTGT								
TuLS23	(TAA)6	F: TGGTAACTGCATCAACCATA	34	3	PET	KJ545965	193–202	4	0.21	0.66
		R: AAGATTCGGGAAGATTAAGG								
TuLS24	(GA)7	F: TGTTGTATGGGAATAAGACAAG	36	3	VIC	KJ545966	225–238	10	0.26	0.69
		R: GTGATTGGCCTGATAATGTT								
TuLS35	(TG)8	F: GGAAACGTATCACAATTTGG	100	2	FAM	KJ545967	204–292	8	0.17	0.70
		R AGAATCTTTTGTTGCTTCCA								
TuLS38	(CAA)6	F: CAACACCAATCACAAAATGA	15	1	NED	KJ545968	239–253	5	0.03	0.13
		R: GTTGGACTTGGTGAATCAGT								
TuLS39	(AGC)6	F: ACATTATCGTTCGGTTCATC	17	3	VIC	KJ545969	270–293	7	0.04	0.15
		R: CTTTGTTCCCTTTTATGTGC								
TuLS41	(CAT)6	F: GAATGAAGATTGGTGGGTTA	23	3	PET	KJ545970	242–261	8	0.27	0.53
		R: TCAAGATTTTGGAATCAGAGA								
TuLS42	(ATC)5	F: TTCCTCTTCCTTGTCTTTCA	27	3	FAM	KJ545971	230–269	10	0.07	0.52
		R: CATCATCTTGTTGTTTGTGC								
TuLS43	(GAT)5	F: AATGGAGGTATGGATGACG	28	3	NED	KJ545972	262–277	4	0.02	0.14
		R: AAAGCTGCTGAAAGTCACTC								
Tupm07*	(CT)10	F: CCAATCACTGTGTTGATCGC	13	3	NED	na	79–94	10	0.43	0.71
		R: GGCTGGTTTCTCTTTCTCCC								
Tupm08*	(AG)10	F AAGCAACAGTTTAGGATGAGAAGG	16	2	PET	na	78–92	8	0.33	0.81
		R: AGTCCATCTTCCTCTTGTCTTCTAGT								
Tupm09*	(CT)10	F: TGAAAAGCGAAACATTGATTCTA	6	3	FAM	na	74–91	8	0.31	0.80
		R: GAAATGTCGAGTTGTCAGGG								
TuCA12*	(CA)7	F: GATTTGTGGTCGTGGTTTTC	9	1	FAM	AB263078	177–287	15	0.23	0.78
		R: GATCAACTCAAAAGGATAACGTTG								
TuCA83*	(GT)6	F: CAGGGTGAAACTTAGATACC	2	1	VIC	AB263081	201–219	11	0.24	0.74
		R: CAATTTTCCCTCTACATCTC								
TuCA96*	(TG)7	F: ATGGATTGTCACCGATTTCA	11	1	PET	AB263082	109–218	4	0.10	0.60
		R: CTGAAGTTTACTTGCTATAGTC								
TuCT09*	(CT)15	F: GATCACTTTTTCATGTTATTCTG	na	2	FAM	AB263084	108–118	7	0.08	0.53
		R: CTTGGAATGAACTTTAGCAC								
TuCT67*	(CT)9	F: CCATCATCTTCATCATTCTTCACC	9	2	NED	AB263090	88–112	15	0.21	0.73
		R: TAGAACAGTCAAGCAAAAAGAGTC								
TuCT73*	(CA)7	F: CGATGTGGGTGGTAAGCATG	18	1	VIC	AB263091	106–214	9	0.25	0.69
		R: ACGATGATATTGATGATGAGCG								
Tu35b*	(TGA)8	F: CTTCCCGAAGGCTGTTGATA	1	1	NED	AJ419832	91–113	6	0.22	0.40
		R: AATGGAATGAGTTATCGTTGGG								

The scaffold number is given as indicated in the annotated whole genome of *T. urticae* and can be retrieved at ORCAE [16].“Na” is the number of alleles. Observed heterozygosities (*H*o) Expected heterozygosities (*H*e) calculated with ADEgenet R package [17].

* Loci published previously.

The microsatellite kits successfully amplified 686 individuals from 60 populations originating from a wide geographical range (localities in the Northern Mediterranean basin), what highlights the potential usefulness for population genetic studies. The number of alleles per locus ranged from 3 to 16 and expected heterozygosity values ranged from 0.12 to 0.81 (Table 1). Most of the loci were not at Hardy–Weinberg equilibrium and showed a significant excess of homozygotes, a feature frequently perceived in field populations of *T. urticae* (e.g. [4, 14]). This can be explained by the biology of *T. urticae*, which is an arrhenotokous species [15] (diploid females produce haploid males from unfertilized eggs) what tends to form new colonies from very small propagule sizes and often from a single mated female. Each microsatellite locus characterized in this paper can be mapped on the *T. urticae* genome, what makes it of particular interest for further quantitative genetics applications.

## Data accessibility

DNA sequences: Genbank accessions KJ545959 to KJ545972; AB263078-AB263081-AB263082-AB263084-AB263090-AB263091 and AJ419832.

Genome data: Individual scaffolds of the *T. urticae* (London) genome are available through GenBank under accession numbers HE587301 to HE587940.

## Abbreviations

MtDNA: Mitochondrial DNA
DOE-JGI: Department of Energy Joint Genome Institute
Mb: Megabase
Kb: Kilobase
Bp: Base pair
PCR: Polymerase chain reaction
µL: Microliter.
